# TGF-ß Regulates Enamel Mineralization and Maturation through KLK4 Expression

**DOI:** 10.1371/journal.pone.0082267

**Published:** 2013-11-20

**Authors:** Andrew Cho, Naoto Haruyama, Bradford Hall, Mary Jo S. Danton, Lu Zhang, Praveen Arany, David J. Mooney, Yassine Harichane, Michel Goldberg, Carolyn W. Gibson, Ashok B. Kulkarni

**Affiliations:** 1 Gene Transfer Core, National Institute of Dental and Craniofacial Research, National Institutes of Health, Bethesda, Maryland, United States of America; 2 Functional Genomics Section, Laboratory of Cell and Developmental Biology, National Institute of Dental and Craniofacial Research, National Institutes of Health, Bethesda, Maryland, United States of America; 3 Global Center of Excellence (GCOE) Program, International Research Center for Molecular Science in Tooth and Bone Disease, Tokyo Medical and Dental University, Tokyo, Japan; 4 Harvard University, Cambridge, Massachusetts, United States of America; 5 Université Paris Descartes, Paris, France; 6 University of Pennsylvania, Philadelphia, Pennsylvania, United States of America; University of Southern California, United States of America

## Abstract

Transforming growth factor-ß (TGF-ß) signaling plays an important role in regulating crucial biological processes such as cell proliferation, differentiation, apoptosis, and extracellular matrix remodeling. Many of these processes are also an integral part of amelogenesis. In order to delineate a precise role of TGF-ß signaling during amelogenesis, we developed a transgenic mouse line that harbors bovine amelogenin promoter-driven Cre recombinase, and bred this line with TGF-ß receptor II floxed mice to generate ameloblast-specific TGF-ß receptor II conditional knockout (cKO) mice. Histological analysis of the teeth at postnatal day 7 (P7) showed altered enamel matrix composition in the cKO mice as compared to the floxed mice that had enamel similar to the wild-type mice. The µCT and SEM analyses revealed decreased mineral content in the cKO enamel concomitant with increased attrition and thinner enamel crystallites. Although the mRNA levels remained unaltered, immunostaining revealed increased amelogenin, ameloblastin, and enamelin localization in the cKO enamel at the maturation stage. Interestingly, KLK4 mRNA levels were significantly reduced in the cKO teeth along with a slight increase in MMP-20 levels, suggesting that normal enamel maturation is regulated by TGF-ß signaling through the expression of KLK4. Thus, our study indicates that TGF-ß signaling plays an important role in ameloblast functions and enamel maturation.

## Introduction

TGF-ß1 is a multifunctional cytokine with important roles in many biological processes including embryonic development, cell proliferation, differentiation and maturation, regulation of the immune system, and extracellular matrix (ECM) synthesis [1-3]. It is expressed during tooth development; particularly in developing and mature ameloblasts [4,5]. TGF-ß signaling is mainly mediated through TGF-ß receptors I and II, which are expressed early in the ameloblasts [5]. Disruption of TGF-ß signaling during early tooth development resulted in accelerated tooth formation in a mandibular explant culture model [6]. In the later stages of tooth development, inhibition as well as activation of TGF-ß signaling resulted in various enamel defects [7-10]. The conventional TGF-ß1 knockout mice showed normal tooth development prior to the onset of multifocal inflammation but displayed dental abnormalities in adult knockout mice when survival was prolonged with dexamethasone [11]. 

Amelogenin, the most abundant protein in the developing enamel matrix, is expressed by the secretory ameloblasts as early as embryonic day 15 (E 15), and continues to be expressed throughout amelogenesis [12,13]. Amelogenin-deficient mice display defective teeth with disorganized and hypoplastic enamel, characteristic of human amelogenesis imperfecta [14]. The absence or changes in other enamel ECM proteins such as enamelin and ameloblastin also led to enamel defects indicating that these enamel-specific proteins are crucial for normal development and mineralization of enamel [15-18]. During the maturation and mineralization of enamel, these ECM proteins are degraded step-wise by matrix metalloproteinase-20 (MMP-20) and Kallikrein 4 (KLK4) to form mature enamel [19,20]. Both MMP-20 and KLK4 knockout mice display enamel defects with hypomineralization resembling an amelogenesis imperfecta phenotype [21-25]. Interestingly, earlier studies implicated TGF-ß in ECM deposition in bone and cartilage by partially modulating the actions of other growth factors such as epidermal growth factor (EGF) on metalloproteinases and tissue inhibitor of metalloproteinases (TIMP) [26]. In vitro studies revealed that TGF-ß1 plays an important role in the maturation-stage enamel organ, in apoptosis of ameloblasts [10], and in the regulation of MMP-20 expression [5]. However, the mechanism by which TGF-ß signaling modulate expression of these proteases to regulate enamel formation or mineralization in vivo has not been investigated. In this report, we used ameloblast-specific TGF-ß receptor II conditional knockout mice to delineate the molecular role of TGF-ß signaling in ameloblast functions during enamel development. 

## Materials and Methods

### Generation of Bovine Amelogenin Promoter-Driven Cre (bAMG-Cre) Mice and TGF-ß Receptor II Conditional Knockout (Tgf-ß R2 cKO) Mice

The transgenic vector bAMG-Cre was engineered using a 7.1 kb bovine amelogenin promoter fragment, which includes non-coding exon 1, intron 1, and an engineered Not I site just prior to amelogenin ATG in exon 2. The promoter was ligated to a 2.1 kb fragment containing the bacteriophage P1 Cre recombinase gene, the amelogenin 3’ UTR (500 bp EcoRI fragment), and the pSV40 polyadenylation site for gene stabilization (Figure S1A). The bAMG-Cre transgene was purified from the vector backbone and was microinjected into FVB/N zygotes as previously described [27]. The founder mice were genotyped by Southern blot analysis (data not shown). The bAMG-Cre line A1 with optimal ameloblast-specific expression was selected for subsequent experiments. The Cre gene in the offspring of the founder line was confirmed by PCR analysis [28]. 

The ameloblast-specific TGF-ß Receptor II conditional knockout mice (TGF-ß-RII F/F; bAMG-Cre+/-, hereafter referred to as “cKO”) were generated by crossing bAMG-Cre mouse line A1 with TGF-ß-RII F/F mice [29]. We used a PCR strategy for detecting wild-type, TGF-ß-RII floxed, and cKO alleles, as described previously ([29]; see Figure S2A and B). All animal studies were conducted in compliance with the NIH guidelines for the Care and Use of Laboratory Animals and approved by the Institutional Animal Care and Use Committee (IACUC) of the National Institute of Dental and Craniofacial Research.

### Functional Analysis of AMG-Cre Mice

The bAMG-Cre mice were crossed with ROSA26R reporter mice to assess the ameloblast specific Cre activity. Molars and incisors were obtained from pups at E18.5, P0, P2, P4, and P7 of age, and embedded in OCT compounds (Sakura Finetek, Torrance, CA). Frozen tooth sections were made at 12 µm thickness using a cryostat (CM3050S, Leica, Buffalo Grove, IL), stained with beta-gal solution (5 mM potassium ferrocyanide, 5mM potassium ferricyanide, 2 mM magnesium chloride, 0.02% NP-40, 0.01% sodium deoxycholate, and 1 mg/ml of Blu-o-gal (Life Technologies, Grand Island, NY) and counterstained with eosin as described in [28].

### Extraction of Genomic Tail DNA and Tooth DNA for PCR Analysis

For tissue-specific genotyping, tail lysates were prepared by adding tail tips to the tissue lysis buffer and proteinase K [30]. Tooth lysates were also obtained from the mouse incisors and molars. Genomic tail and tooth DNAs were extracted from the lysates by the standard phenol-chloroform extraction and ethanol precipitation method and used for PCR analysis for TGF-ß-RII alleles and Cre as described above. 

### Histology and Immunohistochemistry

The cKO and the control mouse skulls at P2 and P7 were fixed in 4% paraformaldehyde and demineralized by EDTA, and 5 µm paraffin sections were cut and stained using hematoxylin and eosin (H&E) or Masson Trichrome for general histology. Immunostaining for TGF-beta receptor II (Santa Cruz Biotechnology, Santa Cruz, CA) amelogenin (Santa Cruz Biotechnology), Ki67 (Dako, Carpentaria, CA) as well as ameloblastin and enamelin [22] was carried out using either Biocore Medical reagents or Vectastain ABC Elite Kit (Vector Labs, Burlingame, CA). The dilutions used for the antibodies for amelogenin, Ki67, TGF-beta receptor II amelogenin, enamelin and ameloblastin were 1:100, 1:200, 1:500, 1:500 and 1:100, respectively. For determination of apoptosis, Terminal deoxynucleotidyl transferase dUTP nick end labeling (TUNEL) assay was performed using in-situ apoptosis detection kit (MK500, Takara Bio Inc., Mountain View, CA) according to the manufacturer’s protocol. 

### Phenotypic Analysis by X-Ray, Scanning Electron Microscopy (SEM), and Microcomputed Tomography (µCT) Analysis

For X-ray and µCT analysis, 1-month- and 3-month-old mouse skulls of control and cKO mice were dissected, cut symmetrically in half, fixed in 4% paraformaldehyde, and stored in phosphate buffered saline (PBS). X-ray analysis was performed using the Faxitron MX20 (Faxitron Bioptics, Tucson, AZ) as described earlier in [8]. Morphological analysis and measurement of enamel mineralization of the mandibular molars were further performed using a cone beam µCT scanner (SMX-100CT; Shimadzu, Kyoto, Japan). The X-ray tube was operated at 75 kV, 200 µA, with brass filter of 0.1 mm thickness. The image scans were performed at a 10 µm/voxel resolution setting. The three-dimensional images were re-constructed and measured by software (TRI/3D-BON software; RATOC System Engineering, Tokyo, Japan). Mineral density values were calculated based on a linear calibration equation determined by hydroxyapatite (HA) standards (Phantom type 2, RATOC System Engineering). The enamel phases were segmented from mandibular bone and dentin with threshold values set at 1750 mg HA/cm^3^, and then mineralized enamel volumes and enamel mineral densities were calculated. Fixed 3 month-old control and cKO molars were used for SEM analysis. SEM samples were etched with 0.1% nitric acid three times for 10 sec each; rinsed in running tap water, and dried and sputter-coated with gold-palladium for SEM as described in [31]. 

### RNA Extraction and Quantitative PCR (qPCR)

The mandibular and maxillary molars of P2 and P7 cKO and the control (F/F) mice were dissected and homogenized with a homogenizer (Precellys 24, Bertin Technologies, Rockville, MD) in Trizol reagent (Life Technologies). Total RNA was isolated using RNeasy Mini kit (Qiagen, Valencia, CA) and 0.5 µg of RNA was used to synthesize complementary DNA (cDNA) using the iScript Reverse Transcription Supermix Kit (Bio-Rad, Hercules, CA). Real time qPCR was performed with amelogenin, ameloblastin, enamelin, MMP-20, KLK4, GAPDH QuantiTect primers and iQ SYBR GreenSupermix (Bio-Rad). Samples were run in quadruplicate for 40 cycles using the Thermal Cycler (CFX96, Bio-Rad). Gene expression was analyzed by adopting the standard method of real-time PCR and results were expressed as a relative fold change in gene expression compared to the control.

### Statistical Analysis

The data were analyzed for statistical significance using Graph Pad Prism, version 5.00, for Windows (Graph-Pad Software, Inc., La Jolla, CA). A two-tailed Student’s unpaired t test was used to determine statistical significance for all comparisons. Values of *P* < 0.05 were considered statistically significant. 

## Results

### Developmental Ameloblast-Specific Cre Expression

Functional validation of the bAMG-Cre line A1 using ROSA26R reporter mice revealed that the ß-galactosidase activity (*LacZ* positive cells, arrows) was specifically localized in ameloblasts. Developmental expression of bAMG-Cre varied from E18.5 to P7 (Figure S1B). In the incisors, the bAMG-Cre activity was noted between P0 and P7 with a peak level at P4, whereas in molars the bAMG-Cre activity was obvious between P2 and P7 with a peak level at P7. The bAMG-Cre activity increased as enamel matrix was being formed and matured in molars. The peak bAMG-Cre activity levels were seen in incisors at earlier stages than that of molar as the incisor development precedes that of molars. At the same developmental stage, the *LacZ* staining intensity generally appeared stronger in molars than in incisors, indicating that the promoter activity used in this Cre line was consistent with a previous report [32]. We have used this bAMG-Cre line A1 line to conditionally knock out TGF-ß signaling through deletion of TGF-ß Receptor II.

### Enamel Mineralization Defects and Attrition in TGF-ß RII cKO Mice

**Figure 1:**
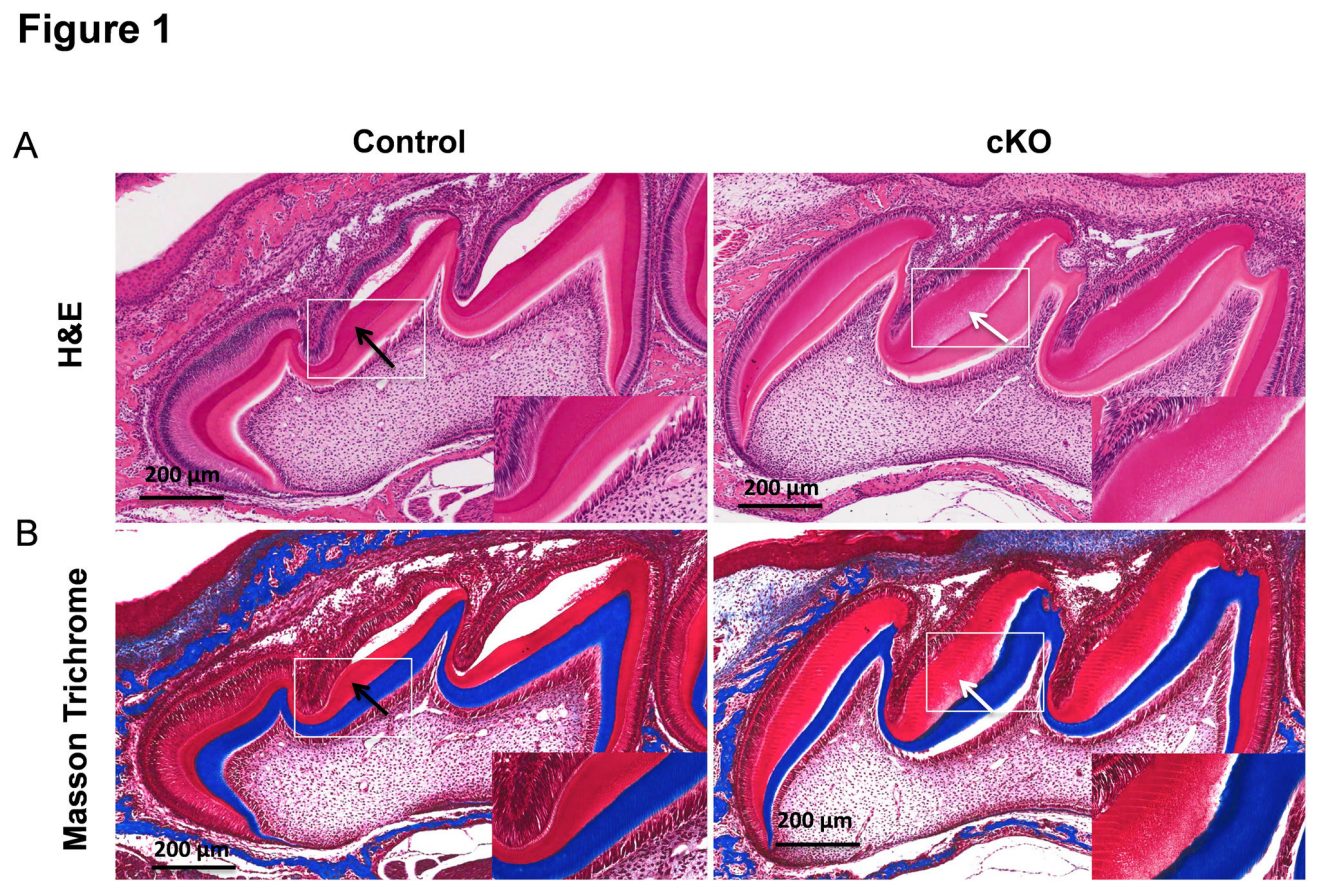
Differential staining pattern of enamel matrix components between control and cKO P7 molars. (A) H&E staining showing dark red staining in enamel matrix in the control molar (black arrow) whereas staining is pale and dispersed within the enamel matrix of the cKO molar (white arrow). (B) Masson Trichrome staining shows similar differential staining pattern in the enamel matrix between the two groups. Bar = 200 µm. Higher magnifications are shown in insets.

We confirmed the successful deletion of TGF-ß-RII alleles in the cKO teeth by PCR and by immunohistochemical analysis (Figure S2C). As shown in lane 6 of the gel pictured in Figure S2B, we detected the expected size amplicon for deletion of the floxed segment of the TGF-beta Receptor II in the cKO teeth, whereas we could not detect similar size amplicon in the tooth DNA from the control mice. The TGF-ß RII staining was found to be significantly lower in cKO ameloblasts (Figure S2C, white arrows) as compared to the control (Figure S2C, black arrows). The histological analysis of the teeth revealed normal ameloblast morphology in the cKO teeth. However, significant differences were observed in the pattern of staining of enamel matrix in the cKO teeth as compared to the controls (Figure 1A and B). The control enamel displayed dark and intense red staining with H&E and Masson Trichrome (black arrows) whereas the cKO enamel showed pale and uneven staining (white arrows) suggesting altered components or protein characteristics in the cKO enamel.

**Figure 2:**
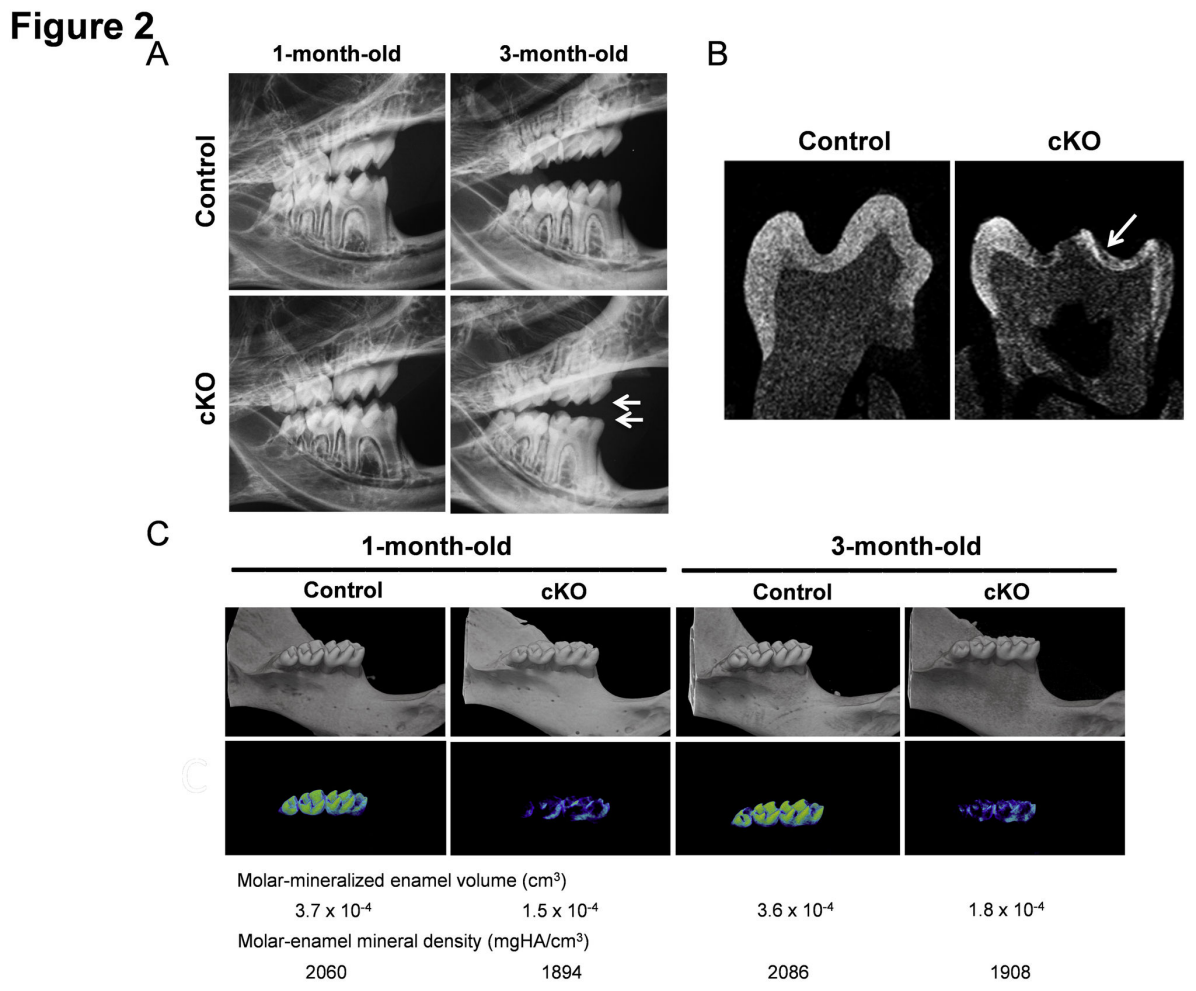
Enamel attrition in the cKO teeth. (A) Radiographs of 1- and 3-month-old mouse teeth. 3-month-old mandibular and maxilla of a cKO mouse shows enamel attrition along the molar cusps (white arrows). (B) µCT images of a single slice view of the molar showing thinner enamel along the molar cusps in a cKO mouse (white arrow). (C) Quantitative µCT analysis of the mandibular molars shows more than two fold decrease in molar mineral enamel volume and a slight decrease in molar enamel density in cKO mice in both 1 and 3 month old samples.

Further analysis of the cKO teeth by x-ray analysis showed dental attrition of the molar cusps in three-month-old mice (Figure 2A, white arrows). The single section images of the mandibular molars by µCT analysis also showed a flattened enamel layer in the three-month-old cKO mice, suggesting that molar enamel acquired mineralization defects resulting in increased attrition with age (Figure 2B, white arrow). The quantitative µCT analysis for molar enamel mineral density (Figure 2C) further confirmed apparent local aberrations with decreased mineralization in the cKO molars. The molar-mineralized enamel volume also showed a significant decrease (more than two fold) in the cKO mice in both one-month- and three-month-old mice when compared to controls (Figure 2C). Although the control enamel could be clearly segmented from bone and dentin at the 1750 mg HA/cm^3^ threshold, the hypo-mineralized enamel in cKO mice could not be clearly separated from bone and dentin. 

**Figure 3:**
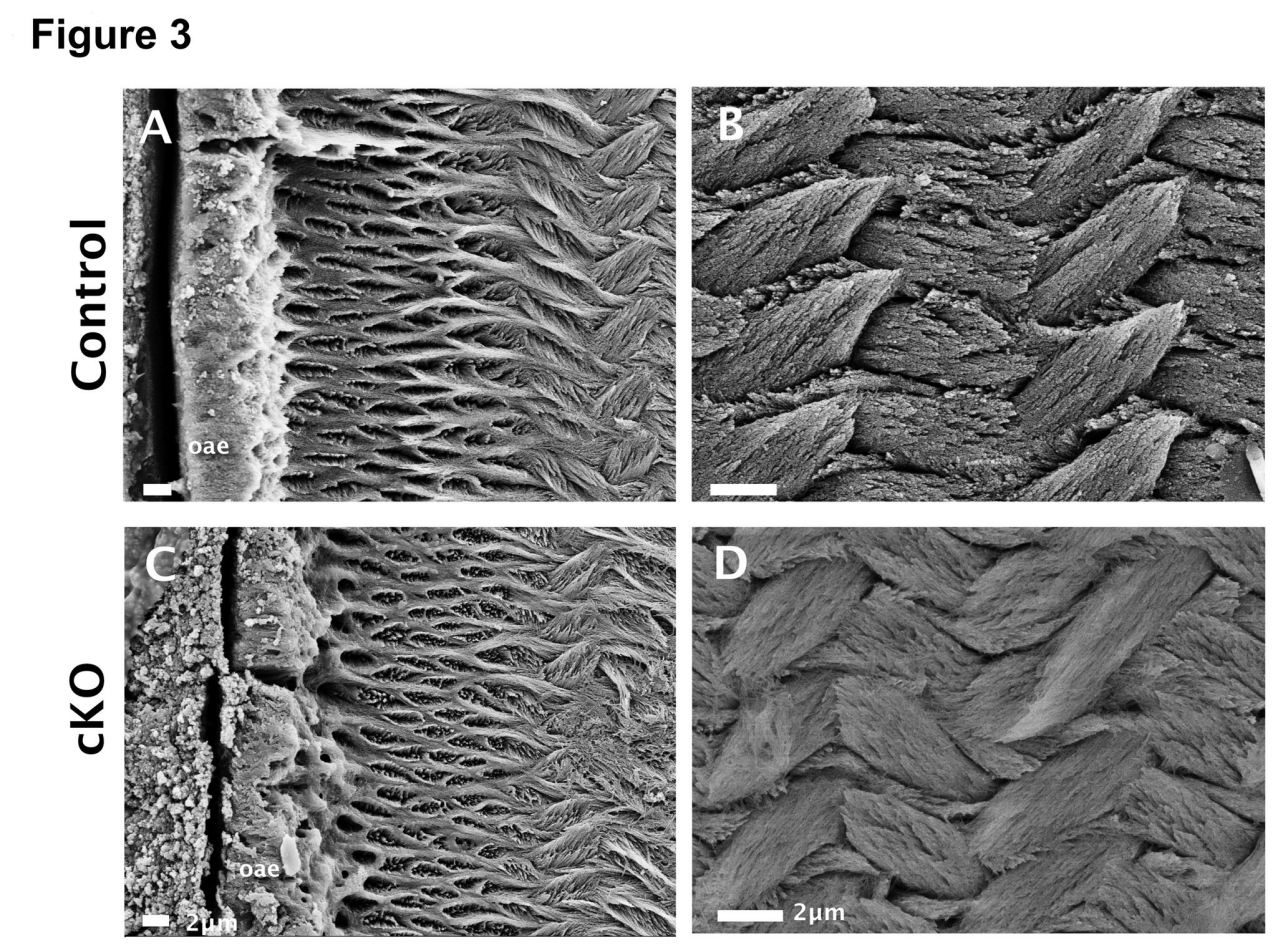
Aprismatic enamel and thinner crystals in cKO mouse enamel. SEM analysis showing irregular and porous outer aprismatic enamel (oae) (panel C, bar = 2 µm) and thinner crystallites in the subjacent prismatic enamel (panel D, bar = 2 µm) as compared to the controls enamel (panels A and B).

The SEM analysis for the molar outer aprismatic enamel (oae) shows irregular organization with increased porosity in the cKO mouse as compared to the control (Figure 3A and C). In the subjacent prismatic enamel, the control shows the normal crystallite thickness and solid structure in rods and interrods; the cKO enamel crystallites are thin and prismatic and look disorganized (Figure 3B and D). Differences in mineralization make the etching pattern more accentuated in the control and less in the cKO enamel. 

### Altered Localization of ECM Proteins in in TGF-ß RII cKO Enamel

**Figure 4:**
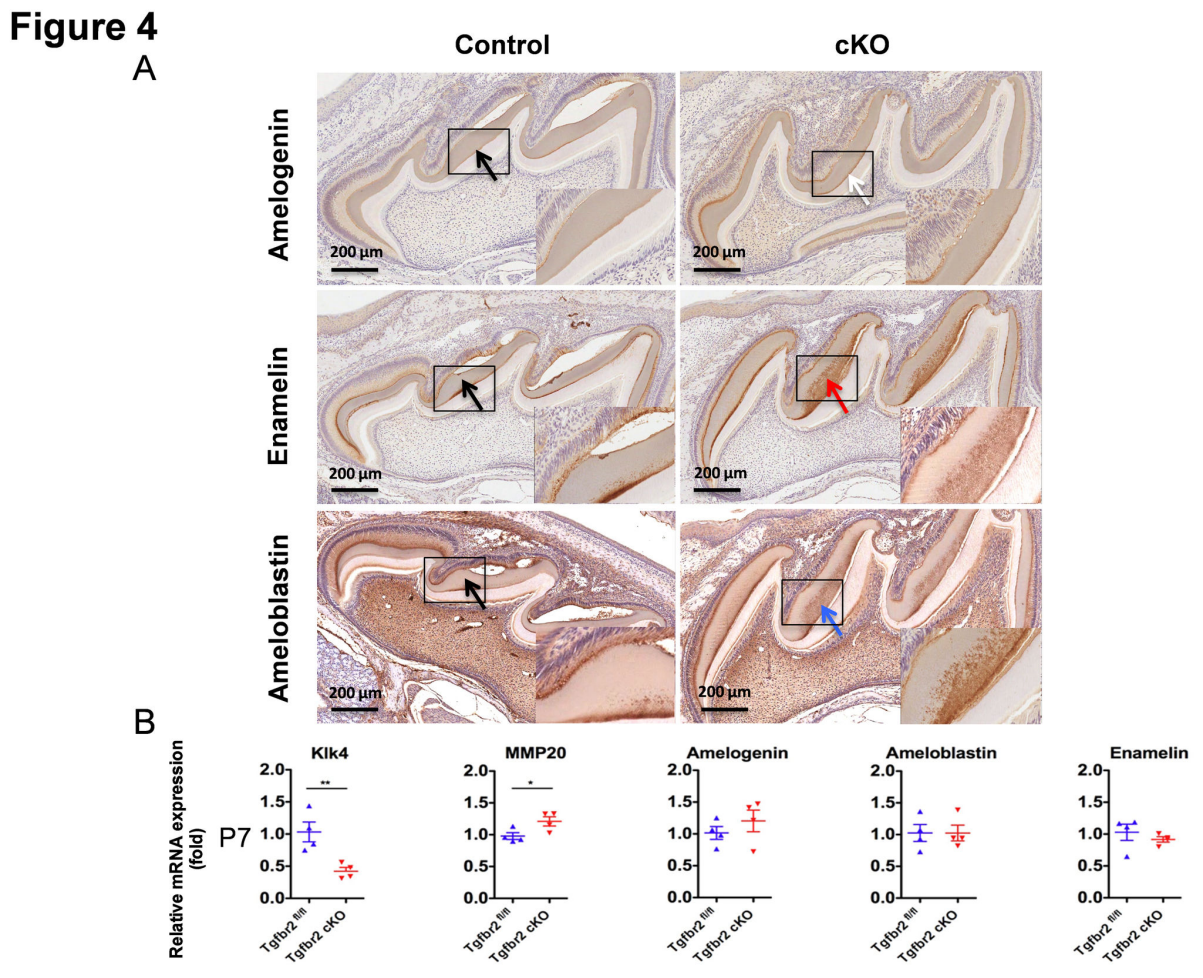
Processing defects of enamel matrix proteins in the cKO mice. (A) Darker and slightly higher amelogenin staining in P7 cKO mouse in the enamel matrix (white arrow) as compared to the control (black arrow), residual enamelin in P7 cKO enamel matrix (red arrow), and higher ameloblastin staining in P7 cKO enamel matrix (blue arrow). Higher magnifications are shown in insets. (B) qPCR showing a significant decrease in KLK4 mRNA expression and slight increase in MMP-20 mRNA expression at P7. No difference in amelogenin, enamelin, and ameloblastin mRNA expression at P7. Values of *P* < 0.05 were considered statistically significant (***P < 0.001 and *P < 0.05).

To investigate the possible cause of tooth defects that led to the dental attrition phenotype in the cKO enamel, we analyzed enamel matrix proteins by immunostaining with specific antibodies. At P7, more intense staining was observed for amelogenin, enamelin, and ameloblastin in the cKO enamel as compared to the control (Figure 4A). To determine how deletion of TGF-ß signaling affects the ameloblasts, we performed qPCR analysis specifically for enamel matrix and enamel processing proteins such as amelogenin, enamelin, ameloblastin, KLK4, and MMP-20 in P2 and P7 molars. MMP-20 and KLK4 expression in the cKO teeth were unaffected at P2 (data not shown), but at P7 we observed a slight increase in MMP-20 and significant reduction in KLK4 expression (Figure 4B). The mRNA levels of amelogenin, enamelin, and ameloblastin were unaltered at both P2 (data not shown) and P7, indicating that the accumulated ECM proteins seen at P7 in the enamel matrix of the cKO mice are mainly due to a decreased KLK4 expression. Because TGF-ß signaling can influence cell proliferation and apoptosis, we performed immunostaining for Ki67, a proliferation marker, as well as TUNEL assay for DNA fragmentation [33] (Figure S3). Ki67 expression was seen only in Hertwig’s epithelial root sheath (HERS) at this stage, demonstrating that there are no obvious effects of TGF-ß down-regulation on ameloblasts proliferation in both control and cKO mice. In both groups, TUNEL staining showed some apoptotic populations in ameloblasts. However, there were no differences between the cKO mice and the controls that could account for any change in the ameloblast population (Figure S3). Therefore, the TGF-ß signaling seems to impact processing enzymes, specifically KLK4, which in turn affects proper processing of the ECM proteins required for a normal bio-mineralization of the enamel. The defects in enamel mineralization result in the weakening of the enamel structure in the form of dental attrition in adult mice.

## Discussion

TGF-ß1, a multi-functioning growth factor, is a key component in proper enamel development as it was shown that TGF-ß1 over-expression in the pre-secretory stage of ameloblasts resulted in an abnormal enamel mineralization pattern [8]. Our TGF-ß-RII cKO mouse did not exhibit a gross tooth phenotype during the early stage of enamel formation. However, when histological analysis was performed on the teeth of P7 mice, we observed different staining patterns in the enamel matrix, indicating altered protein components therein. In adult mice, we observed tooth attrition, presumably caused by altered enamel mineralization or maturation as seen by X-ray, µCT, and SEM analysis, which showed a significant reduction in the molar-mineralized enamel volume and density. TGF-ß and activin receptors regulate Smad3-mediated downstream signaling and an earlier report on Smad3 null mice showed reduced enamel mineralization, structural changes in the enamel, and residual enamel matrix proteins in decalcified tissue sections [7]. The phenotypic changes in Smad3 null mice were similar to those seen in our cKO mice. These results support that TGF-ß signaling regulates enamel mineralization or maturation at later stages of development.

Interestingly, the changes in enamel crystallites observed in our cKO mice were also similar to those found in KLK4 null mice which displayed normal enamel thickness but rapidly abraded enamel following weaning [22]. However, the cKO mice differ from the MMP-20 null mice which showed malformed enamel that was separated from the dentin [24]. These various enamel defects shown in Smad3 and KLK4 null mice suggest that there might be associations between TGF-ß signaling and enamel proteases. Prior to tooth eruption, enamel proteins are digested by secreted proteases and reabsorbed by the ameloblasts. The two extracellular proteases involved in the cleavage of enamel proteins are MMP-20 and KLK4 [22]. MMP-20 is an early enamel matrix protein-processing enzyme, which initiates protein processing early in the secretory stage, while KLK4 is induced at the later maturation stage, where they play a substantial role during enamel development by degrading the enamel proteins. MMP-20 has been shown to catalyze amelogenin and ameloblastin processing in the enamel secretory stage, which can be observed in molars between P2 and P5. KLK4 catalyzes amelogenin degradation in the transition and early maturation stage of enamel, which can be observed in molars between P6 and P11 [23]. The MMP-20 null molars have irregular enamel layers that vary in thickness at P5 while the KLK4 null molars are similar to that of wild-type mice during the secretory stage [20]. The KLK4 null molar shows residual proteins throughout the enamel layer at day 15, which is near tooth eruption. The different staining patterns in our control and cKO enamels when stained with H&E and Masson Trichrome led us to speculate that TGF-ß signaling targets either enamel matrix structure proteins such as amelogenin, enamelin, and ameloblastin, or the enamel matrix processing proteases such as MMP-20 and KLK4, resulting in the altered composition of enamel matrix proteins, altered pH in the matrix, and mineralization/maturation of the enamel. 

Therefore, we have analyzed amelogenin, enamelin, and ameloblastin by immunostaining on tooth sections from both the secretory (P2) and maturation (P7) stages. The cKO enamel showed increased amelogenin, enamelin, and ameloblastin protein localization at P7, but not at P2, suggesting that the increased residual matrix proteins are present in cKO enamel. To correlate TGF-ß signaling and the ameloblast specific downstream targets, including the potential roles of MMP-20 and KLK4 in the localization of these enamel matrix proteins, we analyzed mRNA expression of representative enamel matrix and protease genes using qPCR analysis. It revealed that in the absence of TGF-ß signaling, the KLK4 mRNA was dramatically decreased during the early maturation stage in the P7 cKO mice, while MMP-20 expression was only slightly increased during the maturation stage. On the other hand, the mRNA levels of enamel matrix proteins were not increased suggesting that TGF-ß signaling does not directly target these structural proteins but it affects their processing through enamel proteases specifically the KLK4 but not so much the MMP-20. This finding is consistent with our cKO model resembling KLK4 null phenotypes but not to those of MMP-20. In addition, in our cKO model, we did not observe a significant relationship between TGF-ß and MMP-20 during the secretory stage of ameloblasts as suggested by earlier in vitro studies [5]. 

The TGF-ß type II receptor interacts with the pro-apoptotic adaptor protein Daxx, which leads to activation of JNK and induction of apoptosis in epithelial cells [34]. TGF-ß acts as an inhibitor of ameloblast growth and a pro-apoptotic factor in ameloblasts in vitro [10]. Moreover, Smad3 mediates the pro-apoptotic effects of TGF-ß by activating caspase-8 [35]. To investigate the reduced TGF-ß signaling on ameloblasts in vivo, we examined ameloblast cell proliferation and apoptosis, which may eventually lead to enamel phenotypes found in the cKO mice. Ki67, a cellular marker for proliferation was strictly localized in the HERS in P7 molars, where the early stage of ameloblasts are dividing and proliferating. However, ameloblasts in secretory or maturating stage did not show Ki67 expression. On the other hand, the TUNEL positive ameloblasts were found in both control and P7 cKO sections, although the number appears to be the same in both genotypes. These results further support that the number of ameloblasts in cKO was not changed, and the enamel phenotypes found in cKO should be the result of altered cellular functions of ameloblasts.

Taken together, we demonstrate that a proper TGF-ß signaling in ameloblasts is important for intricately orchestrated processing of ameloblast-specific proteins for the proper enamel formation and development. 

## Supporting Information

Figure S1 **Generation of amelogenin-Cre mice (bAMG-Cre) and functional validation of line A1 using ROSA26R reporter mice.** (A) Schematic diagram of the amelogenin-Cre construct showing 7.1 kb upstream bovine amelogenin promoter region fused with 2.1kb functional Cre gene, amelogenin 3’ UTR region and SV40pA (B) Ameloblast-specific LacZ expression in incisors and molars during tooth development between E18.5 and P7. Amelogenin promoter driven Cre activities are seen between P0 and P7 in incisors and P2 and P7 in molars (black arrows). The highest Cre activity is at P4 for incisors and at P7 for molars. (a: ameloblast; e: enamel; d: dentin; o: odontoblast; p: pulp).
(TIF)

Figure S2 **Generation of Tgf-ß R2 cKO mice showing abrogation of TGF-ß signaling in ameloblasts.** (A) Schematic diagram showing strategy to detect wild-type, floxed Tgf-ß R2, and cKO alleles using PCR primers P1, P2, P3 and their expectant PCR products; (B) PCR analysis demonstrating generation of Tgf-ß R2 cKO mouse (Lane 6). Lane 1: 100 bp marker, Lanes 2 and 5: tail and tooth DNAs of floxed Tgf-ß R2 control mice, Lanes 3 and 6: tail and tooth DNAs of cKO mice, Lanes 4 and 7: tail and tooth DNAs of WT mice, Lanes 2-4: PCR performed with primers 1, 2, and 3, Lanes 5-7: PCR performed with primers 1 and 3 only. (C) Immunohistochemistry of TGF-ß receptor II demonstrating knocked down expression of TGF-ß signaling in ameloblasts. (a: ameloblast; e: enamel; d: dentin).
(TIF)

Figure S3 **Proliferation and apoptosis assays in ameloblasts and surrounding tooth tissue of P7 control and cKO mice.** In both control and cKO teeth, Ki67 expression is seen only in HERS (Hertwig’s epithelial root sheath) demonstrating that there are no effects of TGF-ß down-regulation on proliferation within the ameloblast layers (number of Ki67 positive cells: 15/53 in control and 16/53 in cKO teeth). TUNEL staining shows some apoptotic activities within the ameloblasts of control and cKO mice, indicating that there are no differential effects of TGF-ß signaling on ameloblasts of control and cKO mice (number of TUNEL positive cells: 47/60 in control and 43/60 in cKO teeth). (a: ameloblast; e: enamel; d: dentin; o: odontoblast; p: pulp).
(TIF)
