# Supplementary figures and images for: TGF-ß Regulates Enamel Mineralization and Maturation through KLK4 Expression
Source: PLoS One. 2013 Nov 20;8(11):e82267. doi: 10.1371/journal.pone.0082267 (PMC3835418; doi:10.1371/journal.pone.0082267)

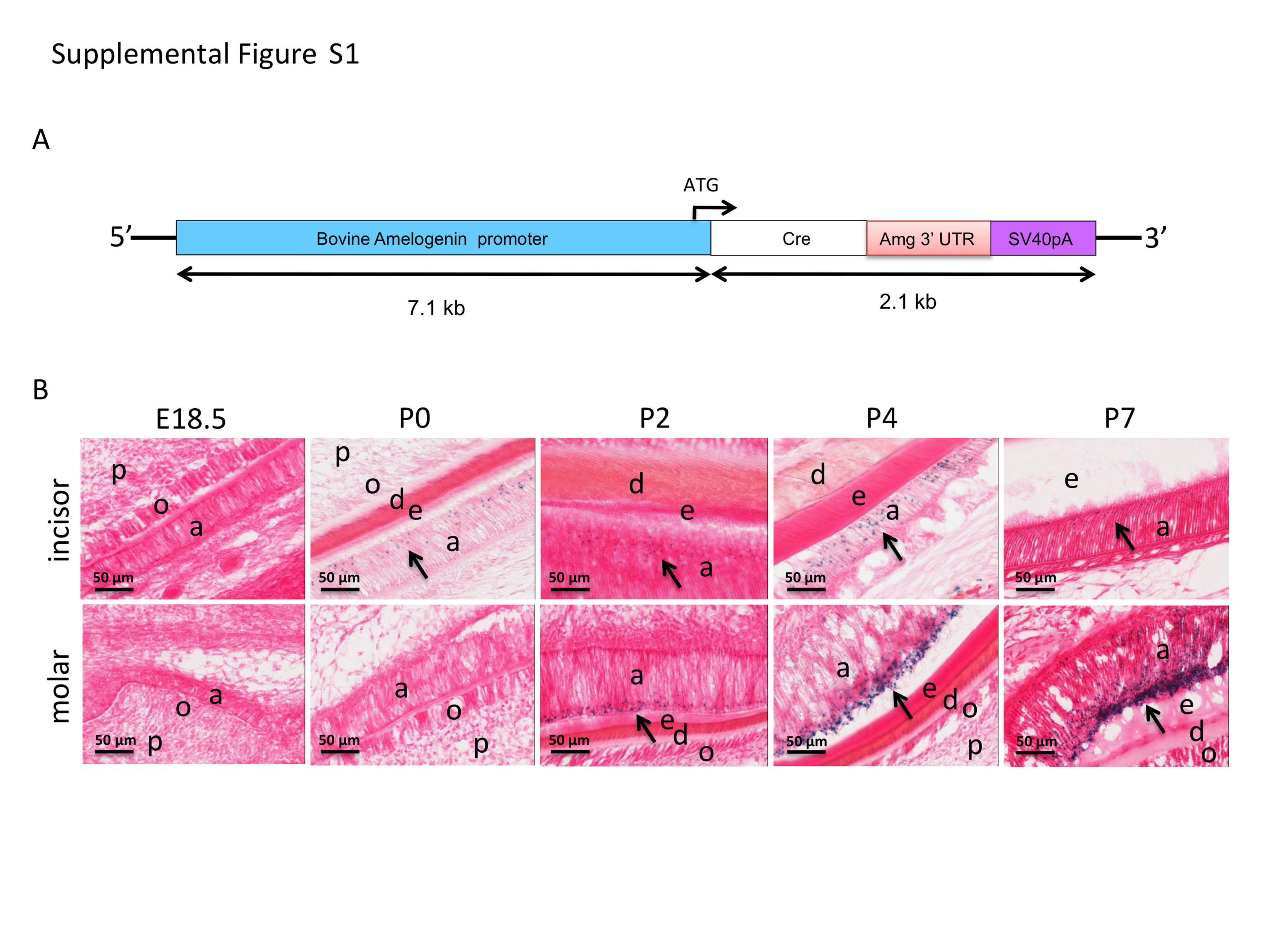

Supplement: Figure S1 — Generation of amelogenin-Cre mice (bAMG-Cre) and functional validation of line A1 using ROSA26R reporter mice. (A) Schematic diagram of the amelogenin-Cre construct showing 7.1 kb upstream bovine amelogenin promoter region fused with 2.1kb functional Cre gene, amelogenin 3’ UTR region and SV40pA (B) Ameloblast-specific LacZ expression in incisors and molars during tooth development between E18.5 and P7. Amelogenin promoter driven Cre activities are seen between P0 and P7 in incisors and P2 and P7 in molars (black arrows). The highest Cre activity is at P4 for incisors and at P7 for molars. (a: ameloblast; e: enamel; d: dentin; o: odontoblast; p: pulp). (TIF) [file pone.0082267.s001.tif]

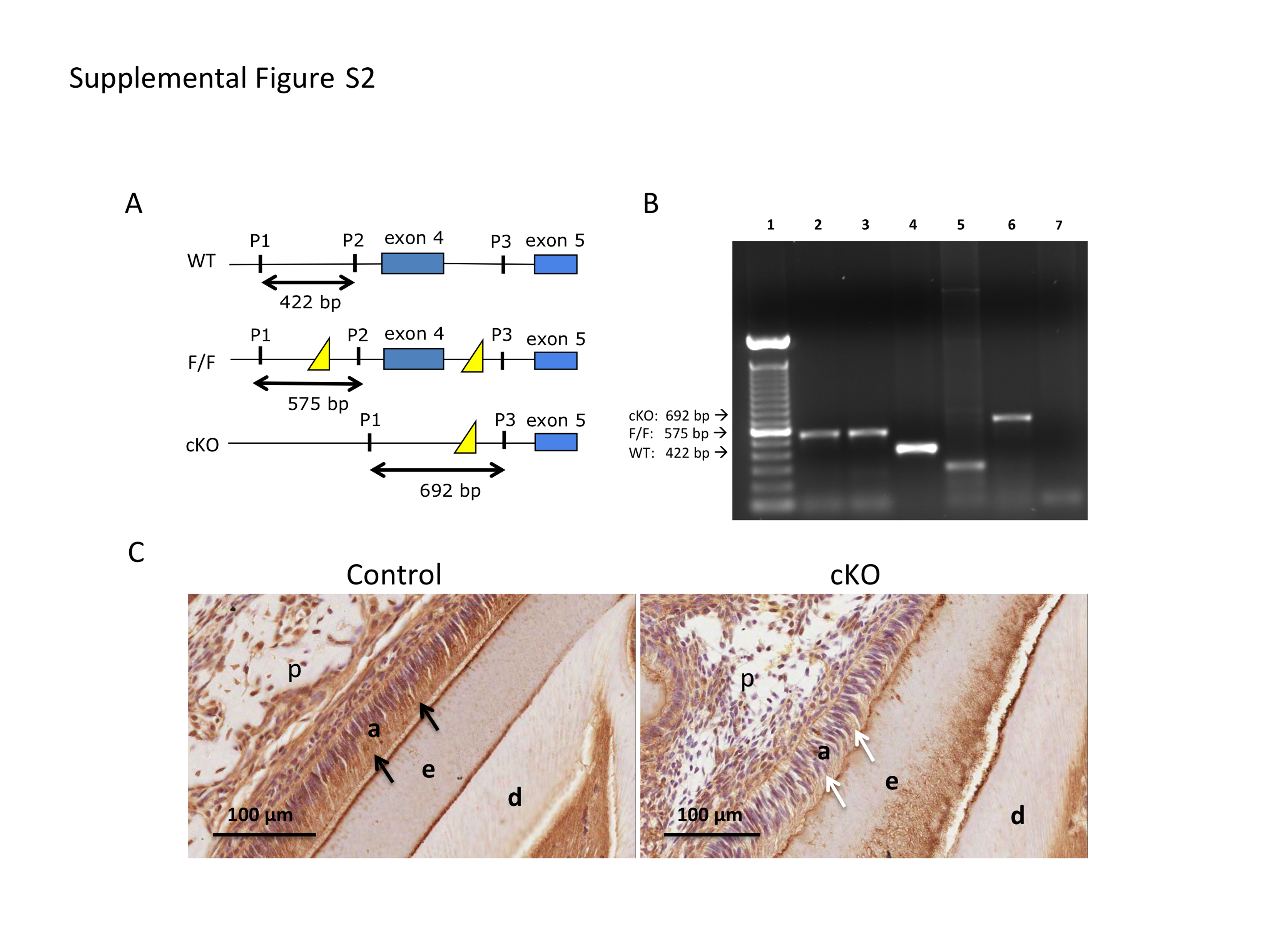

Supplement: Figure S2 — Generation of Tgf-ß R2 cKO mice showing abrogation of TGF-ß signaling in ameloblasts. (A) Schematic diagram showing strategy to detect wild-type, floxed Tgf-ß R2, and cKO alleles using PCR primers P1, P2, P3 and their expectant PCR products; (B) PCR analysis demonstrating generation of Tgf-ß R2 cKO mouse (Lane 6). Lane 1: 100 bp marker, Lanes 2 and 5: tail and tooth DNAs of floxed Tgf-ß R2 control mice, Lanes 3 and 6: tail and tooth DNAs of cKO mice, Lanes 4 and 7: tail and tooth DNAs of WT mice, Lanes 2-4: PCR performed with primers 1, 2, and 3, Lanes 5-7: PCR performed with primers 1 and 3 only. (C) Immunohistochemistry of TGF-ß receptor II demonstrating knocked down expression of TGF-ß signaling in ameloblasts. (a: ameloblast; e: enamel; d: dentin). (TIF) [file pone.0082267.s002.tif]

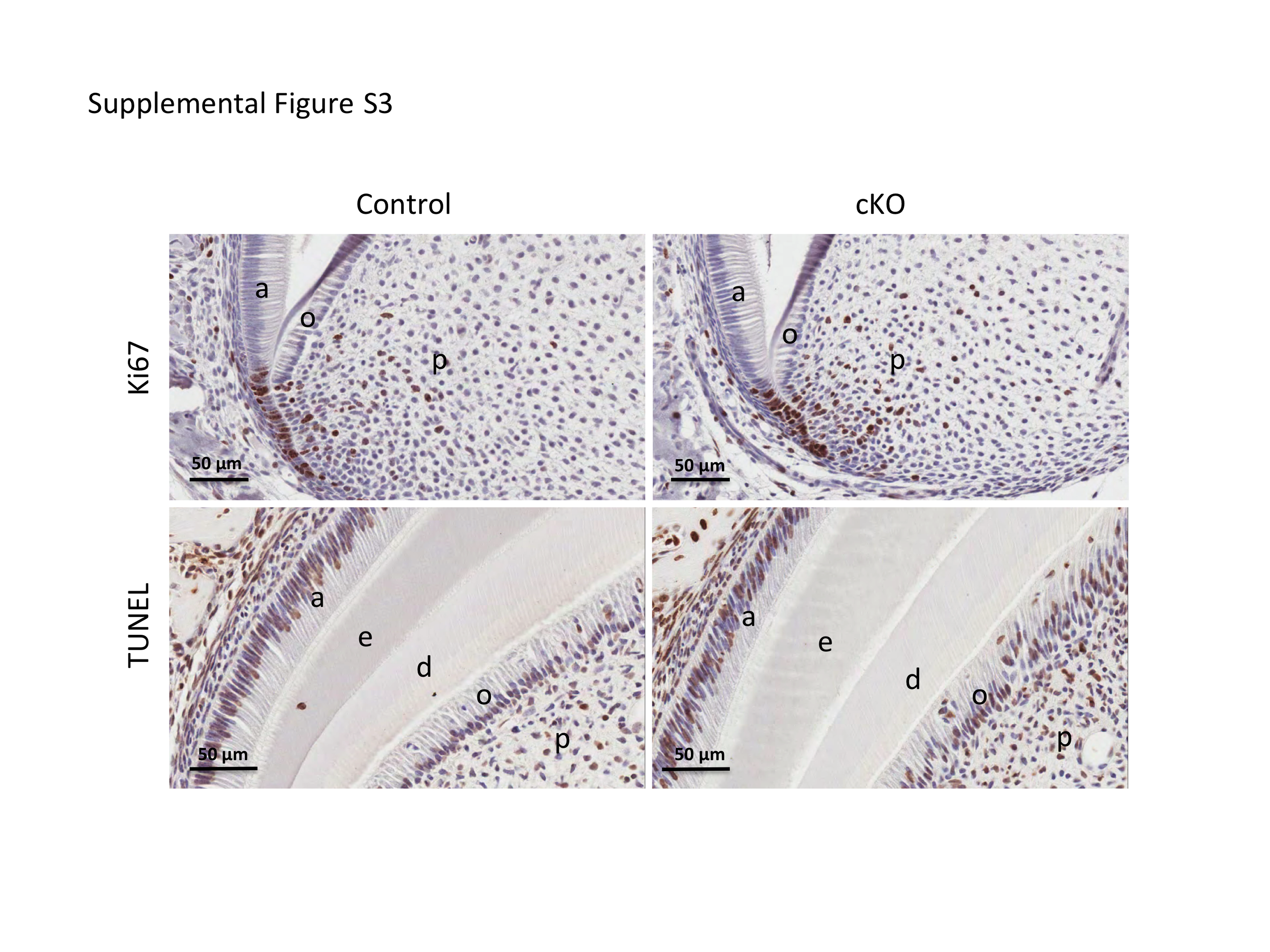

Supplement: Figure S3 — Proliferation and apoptosis assays in ameloblasts and surrounding tooth tissue of P7 control and cKO mice. In both control and cKO teeth, Ki67 expression is seen only in HERS (Hertwig’s epithelial root sheath) demonstrating that there are no effects of TGF-ß down-regulation on proliferation within the ameloblast layers (number of Ki67 positive cells: 15/53 in control and 16/53 in cKO teeth). TUNEL staining shows some apoptotic activities within the ameloblasts of control and cKO mice, indicating that there are no differential effects of TGF-ß signaling on ameloblasts of control and cKO mice (number of TUNEL positive cells: 47/60 in control and 43/60 in cKO teeth). (a: ameloblast; e: enamel; d: dentin; o: odontoblast; p: pulp). (TIF) [file pone.0082267.s003.tif]
